# Comparison of intraoperative handling and wound healing between (NEOSORB® plus) and coated polyglactin 910 suture (NEOSORB®): a prospective, single-blind, randomized controlled trial

**DOI:** 10.1186/s12893-018-0377-4

**Published:** 2018-07-06

**Authors:** Bum Sik Tae, Ju Hyun Park, Jung Kwon Kim, Ja Hyeon Ku, Cheol Kwak, Hyeon Hoe Kim, Chang Wook Jeong

**Affiliations:** 1 Department of Urology, Korea University Ansan Hospital, Ansan, South Korea; 2 Department of Urology, Seoul Metropolitan Government Seoul National University Boramae Medical Center, Seoul National University Hospital, Seoul, South Korea; 3 Department of Urology, Seoul National University Bundang Hospital, Gyeonggi-do, Republic of Korea; 4 Department of Urology, Seoul National University Hospital, 101 Daehak-ro, Jongno-gu, Seoul, 110-744 South Korea

**Keywords:** Surgical site infection, Intraoperative handling, Chlorhexidine acetate, Polyglactin 910 suture

## Abstract

**Background:**

Coated polyglactin 910 suture with chlorhexidine (NEOSORB® Plus) has recently been developed to imbue the parent suture with antibacterial activity against organisms that commonly cause surgical site infections (SSI). This prospective, single-blinded, randomized trial, was performed to compare the intraoperative handling and wound healing characteristics of NEOSORB® Plus with those of the traditional polyglactin 910 suture (NEOSORB®) in urologic surgery patients.

**Methods:**

Patients (aged 19 to 80 years, *n* = 100) were randomized in a 1:1 ratio for treatment with either NEOSORB® Plus or NEOSORB®, and stratified into an open surgery or a minimally invasive surgery group. The primary endpoint was the assessment of overall intraoperative handling of the sutures. Secondary endpoints included specific intraoperative handling measures and wound healing characteristics. Wound healing was assessed at one and 11 days after surgery. Cumulative skin infection, seroma, and suture sinus events within 30 days after surgery were also evaluated.

**Results:**

A total of 96 patients were included, with 47 patients in the NEOSORB® Plus group and 49 patients in the NEOSORB® group. Scores for intraoperative handling were favorable and were not significantly different between the two suture groups. Wound healing characteristics were also comparable. The incidence of adverse events was 13.6%, although none were deemed attributable to the suture, and no difference was observed between the two groups.

**Conclusions:**

NEOSORB® Plus is not inferior to traditional sutures in terms of intraoperative handling and wound healing, potentially making NEOSORB® Plus a beneficial alternative for patients at increased risk of SSI.

**Trial registration:**

ClinicalTrials.gov: NCT02431039. Trial registration date 14 August 2015.

**Electronic supplementary material:**

The online version of this article (10.1186/s12893-018-0377-4) contains supplementary material, which is available to authorized users.

## Background

Surgical site infections (SSI) are the most common hospital-acquired infections among surgical patients [1]. The occurrence of SSI is generally influenced by the patient’s characteristics and underlying conditions, as well as the type of surgery. Furthermore, some reports have suggested that the suture knot may be a central repository for bacteria that contaminate surgical wounds, as it provides a nidus or scaffold for bacterial colonization and replication that may lead to SSI [2]. Therefore, prevention of bacterial colonization at the surgical site with a coated antibacterial suture may help to reduce the incidence of SSI. While there have been many attempts to reduce SSI risk by coating the suture material with various antibacterial components, these products have not demonstrated verifiable performance in clinical trials, with the exception of a few.

Hence, to more effectively reduce the risk of suture contamination and SSI, suture materials were developed that were coated with more powerful antibacterial agents such as triclosan (polychloro phenoxy phenol), a broad-spectrum antimicrobial that is active against both gram-positive and gram-negative bacteria [3, 4]. Since 2002, when antimicrobial PGLA910 (VICRYL® Plus, Ethicon, NJ, USA) was first approved by the United States Food and Drug Administration, a variety of triclosan-coated sutures have been licensed and used widely, including triclosan-coated poliglecaprone antimicrobial suture and triclosan-coated polydioxanone antimicrobial suture [5]. Although considered safe and effective for over 30 years [2], some studies have reported the development of bacteria-resistant strains to triclosan [6, 7]. Therefore, there is a growing need for novel alternative substances such as chlorhexidine (CHX), which is known to infuse surgical sutures with powerful antimicrobial activity.

NEOSORB® Plus (Samyang Biopharmaceutical) is manufactured using heat-treatment technology and biodegradable polymers. In contrast to traditional sutures, NEOSORB® Plus is coated with CHX acetate, which has antibacterial activity against the most common pathogens that likely cause SSI. Moreover, NEOSORB® Plus is designed so that CHX is released as slowly as possible after implantation. The use of CHX specifically distinguishes it from other triclosan-coated sutures, such as Vicryl Plus®, and CHX is an antibacterial agent that is active against both gram-positive and gram-negative bacterial strains, as well as fungi in a dose-dependent manner [8]. It is used principally for its antiseptic and disinfectant action on wounds, in several products for oral protection, and in many dentistry applications [9–11]. T. Koburger et al. compared the antimicrobial efficacy of the antiseptics PVP-iodine, triclosan, CHX, octenidine, and polyhexanide, all of which are currently utilized for pre-surgical antisepsis and for the antiseptic treatment of skin, wounds, and mucous membranes based on internationally accepted standards [12]. The study showed that CHX and triclosan are effective agents with equally low maximum values for prolonged contact time. In terms of immediate effect, CHX is more effective than triclosan. CHX also demonstrates low mammalian toxicity based on pharmaceutical testing, and binds strongly to mucosa and skin [13]. These results suggest that the coated PGLA910 suture with CHX may have extensive utility as an antibacterial suture. We assumed that the CHX-coated PGLA910 suture would be more effective than conventional sutures in terms of wound healing and SSI prevention in patients undergoing a variety of surgeries. However, since the CHX-coated PGLA910 suture is a novel formulation and has not yet been used on humans, it is important to demonstrate its safety and feasibility. In this prospective, a single-blinded randomized controlled trial was undertaken, whose aim was to demonstrate the safety and efficacy of this suture and to establish that the effectiveness of the new suture does not fall below a pre-stated non-inferiority margin (alternative hypothesis). In addition, we sought to prove that the coated PGLA910 suture with CHX does not negatively impact wound healing compared to the conventional coated suture.

## Methods

### Trial design and participants

**Fig. 1:**
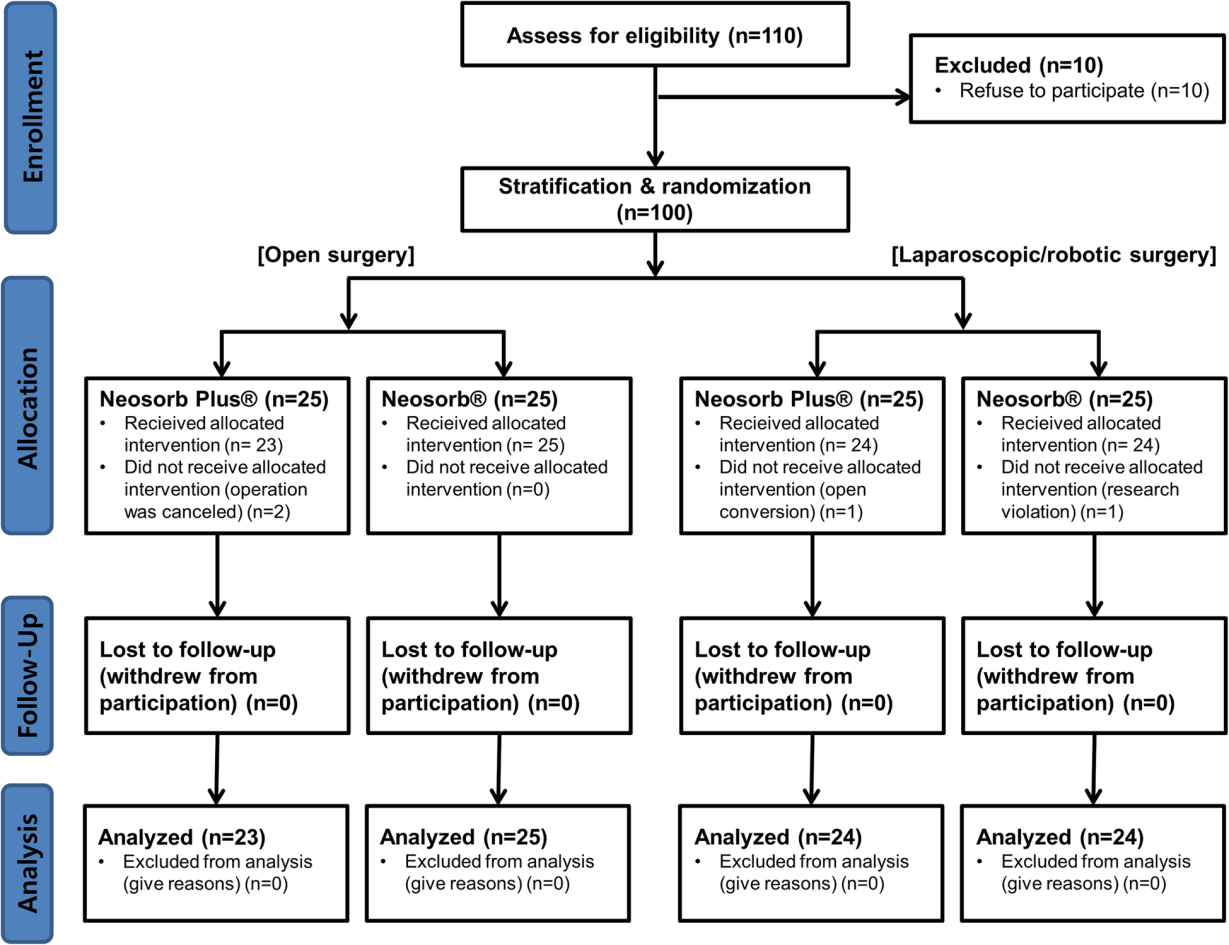
CONSORT participant flow diagram

We designed a prospective, single-blind, randomized controlled, non-inferiority trial at a single center institution comparing the intraoperative handling of suture materials and wound healing outcomes in urologic surgery requiring closure of the fascia and subcutaneous tissue. The trial was registered on the ClinicalTrial.gov database as NCT02431039. We strictly followed the 2010 CONSORT statement to design and report this trial [14, 15]. A total of 110 patients were screened, and 100 patients (aged 19 to 80 years) were enrolled and randomly assigned to the NEOSORB® Plus group (*n* = 50) or a control (NEOSORB®) group (*n* = 50) at a 1:1 ratio according to the type of surgery (open surgery or minimally invasive [laparoscopic or robotic] surgery) as a stratification factor. The flow chart of patient enrollment, allocation, and follow-up is shown in Fig. 1. The inclusion criteria were as follows: age 19 to 80 years, clean or clean-contaminated surgery, urologic surgery requiring closure of the fascia and subcutaneous tissue, and participants who voluntarily signed our clinical trial agreement.

The exclusion criteria included the following: contaminated surgery; wounds requiring retention suture; suspected malnutrition status; active infection status or AIDS; incision sites prone to expand, stretch, distend, or require support; allergy or hypersensitivity to CHX; and any significant active medical illness which, in the opinion of the investigator, would preclude protocol treatment.

### Interventions

All wounds in this study consisted of deep incisions that involved deep soft tissues, muscle, and fascia. The test suture was NEOSORB Plus® and the control suture was the conventional coated PLGA910 suture (NEOSORB®). Surgeries were performed using the standard urological surgery approach for either open or minimally invasive surgery. According to the randomization protocol, the patient’s fascia and subcutaneous tissue repairs were closed with either CHX-coated or non-CHX coated absorbable sutures. Various suture sizes were employed for both the test and control sutures. Wound closure was achieved using routine urologic procedure techniques (subcuticular suture). Postoperative wound dressing was performed once every two days starting at postoperative day (POD) 1.

### Outcomes

Six surgeons (JHP, JKK, JHK, CK, HHK, and CWJ) assessed the intraoperative suture handling characteristics. The primary endpoint was the surgeon’s assessment of the overall intraoperative handling characteristics of each type of suture. Secondary endpoints included the assessment of wound healing and of specific intraoperative suture handling characteristics. The intraoperative suture handling characteristics evaluated included the following: ease of passage through the tissue, first throw knot holding, knot tie-down smoothness, knot security, surgical hand memory, and degree of fraying (Table 1). The handling characteristics of all test sutures were rated on a five-point scale as follows: 1 = excellent; 2 = very good; 3 = good; 4 = fair; and 5 = poor. Wound healing assessments included the healing progress, skin temperature, and the presence of infection, edema, erythema, seroma, and suture sinus (Table 2). Wound healing was evaluated at one (+ 1) day (POD1) and 11 (±4) days (POD11) following surgery. The cumulative events of skin infection, seroma, and suture sinus were again evaluated 30 days postoperatively (POD30).

**Table 1:** The handling characteristics of all test sutures were rated on a five-point scale as follows: 1 = excellent, 2 = very good, 3 = good, 4 = fair, and 5 = poor

Primary endpoint	
Overall handling	The composite evaluation of the suture on all rated characteristics.
Secondary endpoints	
Ease of passage	The ease with which a suture passes through the tissue into which it is being implanted.
First-throw knot holding	Holding opposing tissue edges together with the first throw.
Knot tie-down smoothness	The capacity of a suture to allow a throw or knot to be tied at some distance from its final location and then slide into place with the next throw.
Knot security	The quality of a suture that allows it to be tied securely with a minimum number of throws per knot.
Surgical hand	The surgeon’s gloved feel or tactile reaction to handling the suture.
Memory	The capacity of a suture to remain relatively free of kinking, curling, and other contortions that may interfere with surgical handling and use.
Lack of fraying	Capacity of the suture to resist shredding or unraveling.

**Table 2:** Wound healing assessments

Parameter	Outcome measurement			
Healing progress, apposition	Complete	Incomplete		
Infection	No	Yes		
Seroma	No	Yes		
Suture sinus	No	Yes		
Erythema	0	1+	2+	3+
	None	Linear redness	Redness < 2 mm	Redness > 2 mm
Edema	0	1+	2+	3+
	None	Slight increase in firmness	Skin dimples with pressure	Tense firmness
Pain	0	1+	2+	3+
	None	With pressure	With touching	Constant
Skin temperature	0	1+	2+	3+
	None	Slight	Definite	Hot or radiating

### Randomization and blinding

A researcher in our department screened and enrolled the study participants, who were assigned to either the intervention or the control group by an online computer-generated randomization sequence. The randomization process was guaranteed and managed exclusively by Seoul National University Hospital Medical Research Cooperation Center (MRCC), which had no role in recruitment. Permuted-block random allocation with varying block sizes was performed. The participants were not informed as to whether they were assigned to the study group or the control group until the end of the study. The blinding could be broken at the end of the study if requested by the patients or caregivers, but the blinding could not be broken during the follow-up period. The revelation of the random allocation to patients by the lead investigator was permitted in the event of an emergency threatening the patient’s safety or health.

### Statistical methods and sample size

Analysis was based on a per-protocol analysis and restricted to the participants who fulfilled the protocol in terms of eligibility, interventions, and outcome assessment. Evidence of sample size calculation was based on the following sequence. To show non-inferiority, the upper limit of the 95% confidence boundary of the difference between the two groups could not exceed 20%. Since our study was an exploratory clinical trial and the primary endpoint was not related to patient survival, we decided to set a non-inferiority margin of 20% by clinical judgment. Considering an 80% power and a one-sided type 1 error of 5%, a total of 100 patients (50 in each group) were required to allow for a 2% dropout rate. Logistic regression analysis was used to evaluate intraoperative suture handling techniques. The difference in wound assessment and adverse events between the two groups was analyzed using the Fisher exact test (two-sided). All statistical analysis was conducted using SPSS® Statistics 21.0. The *P* value was considered statistically significant if less than 0.05.

## Results

### Patient population

A total of 100 patients were enrolled and randomized in this study. Four patients withdrew from the trial, leaving 47 patients in the NEOSORB® Plus group and 49 patients in the control (NEOSORB®) group, for a total of 96 treated patients (Additional file 1). Of the four patients who withdrew, two were removed from the study because their surgery was canceled just prior to anesthesia. One patient withdrew because of a change in the operation plan during surgery, and the fourth patient dropped out because of a research violation. Thus, 96 patients overall provided the basis for examining the baseline data, safety assessments, the primary endpoint of overall intraoperative handling, and the secondary endpoints of specific intraoperative handling measurements. Baseline characteristics were similar between the two treatment groups (Table 3). Patients were divided into either an open surgery group (*n =* 48) or a minimally invasive surgery group (*n =* 48) depending on the type of surgery received, as a means of stratification.

**Table 3:** Baseline characteristics of the patients

	NEOSORB Plus (*n* = 47)	NEOSORB (n = 49)	*p*
Age (years)	59.6 ± 12.16	56.16 ± 14.00	0.21
Gender			0.50
Men	35 (74.5%)	35 (71.4%)	
Women	12 (25.5%)	14 (28.6%)	
History of other disease			
HTN	15 (34.5%)	13 (23.4%)	0.32
DM	6 (16.7%)	10 (20.4%)	0.36
Vascular Disease	3 (6.4%)	0 (0%)	0.12
Target organ for surgery			0.53
Kidney	26 (55.3%)	26 (53.1%)	
Bladder	2 (4.2%)	4 (8.2%)	
Prostate	16 (34.0%)	15 (30.6%)	
Other	3 (6.4%)	4 (8.2%)	
Length of incision (cm)	13.30 ± 4.49	14.75 ± 3.90	0.55

*HTN* hypertension, *DM* diabetes mellitus

### Primary endpoint

**Fig. 2:**
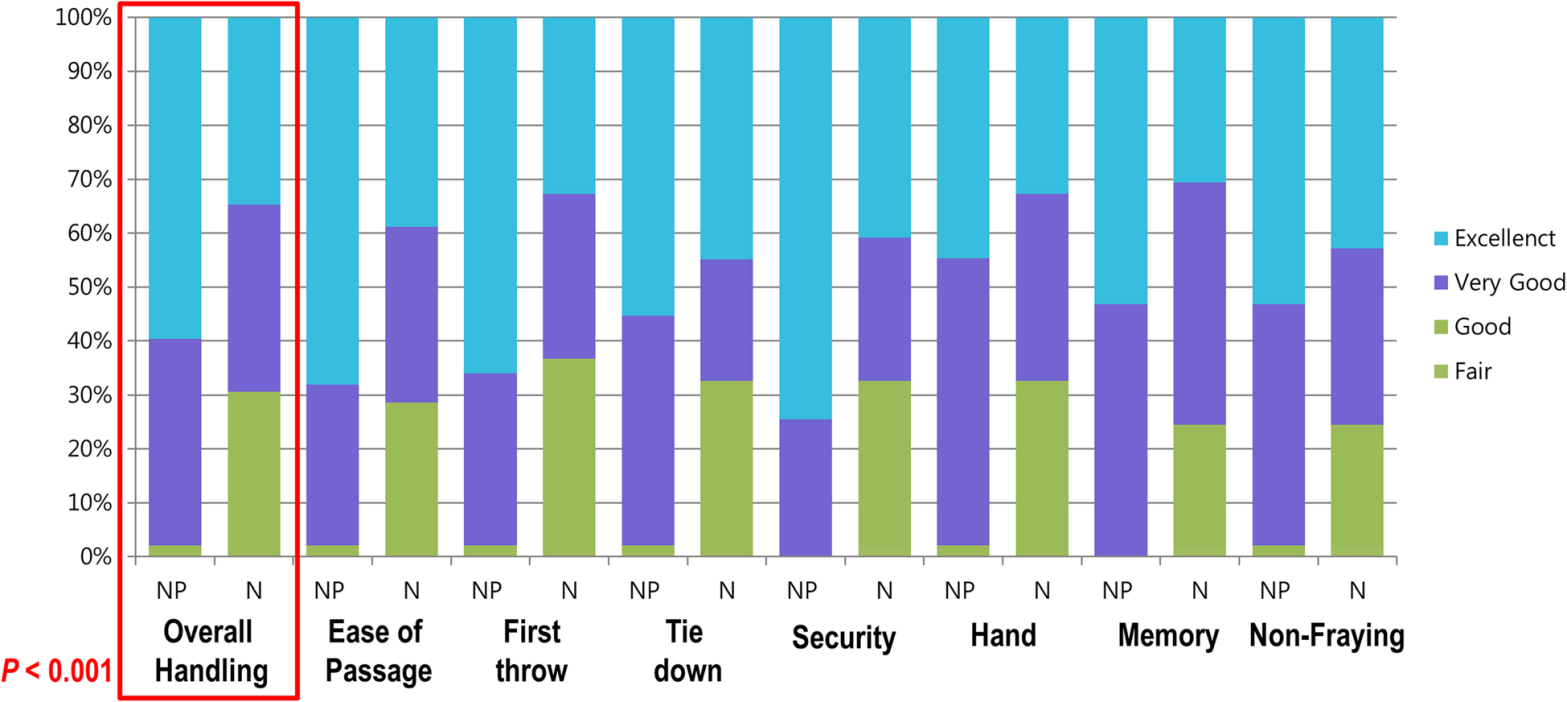
Intraoperative handling. Ninety-six patients (NP *=* 47, *N =* 49) completed the study and were included in the final analysis. The primary endpoint of overall intraoperative handling is shown in the first set of bars. Secondary endpoints for individual aspects of intraoperative handling comprise the remaining bars. Values for good, fair, and poor handling were small and were combined into one measurement. NP, NEOSORB® Plus; N, NEOSORB®

**Fig. 3:**
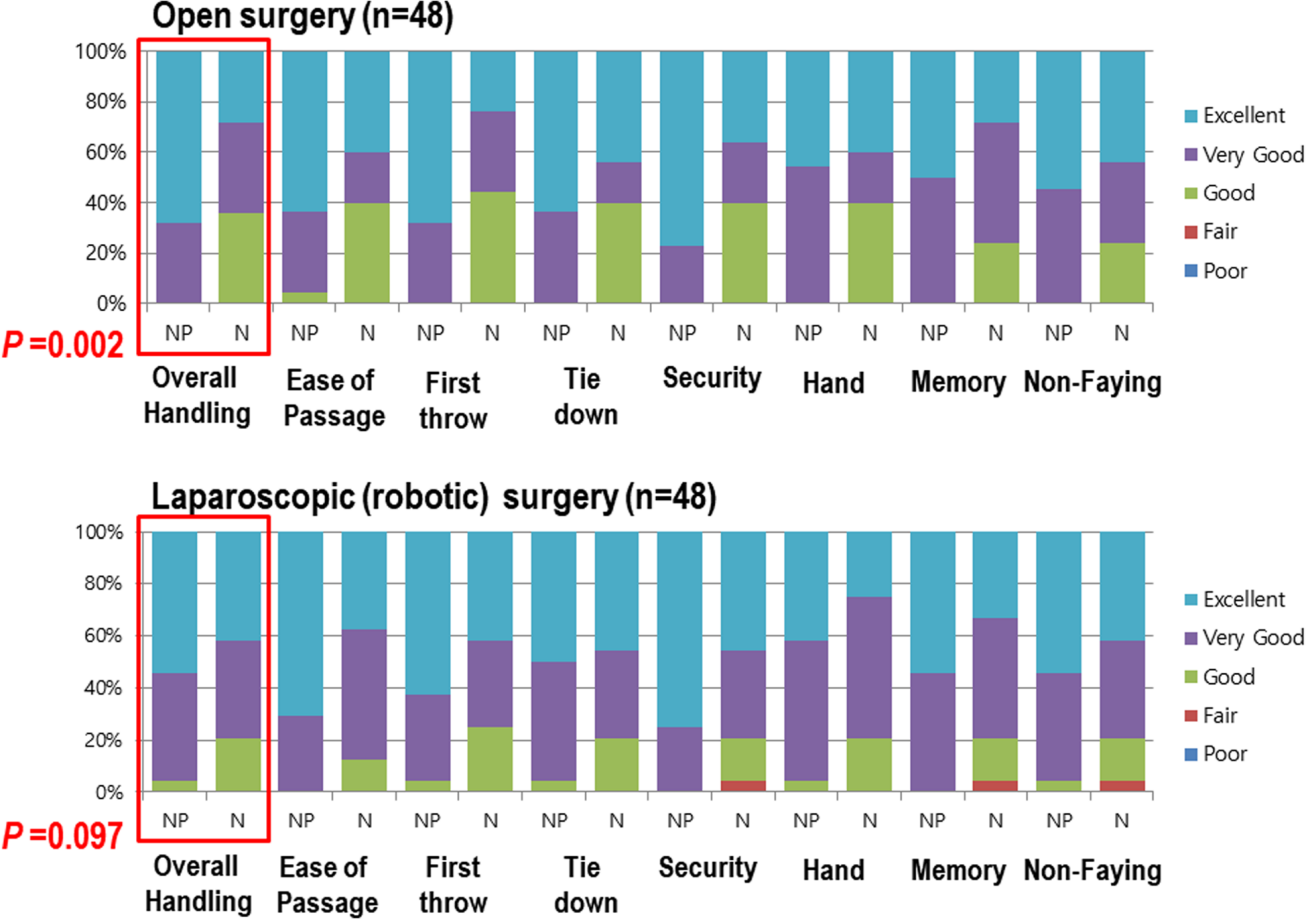
Patients stratified by surgery type. NP, NEOSORB® Plus; N, NEOSORB®

Regarding the primary endpoint, 97.8% of the responses rated the handling as “very good” or “excellent” for NEOSORB® Plus and the difference between groups was statistically significant (*P <* 0.001) (Fig. 2). In the open surgery group, “very good” or “excellent” overall intraoperative handling scores were recorded for NEOSORB® Plus in 100.0% of cases, compared with a mean of 64.0% of cases for NEOSORB® (*P =* 0.001). In the minimally invasive surgery group, “very good” or “excellent” scores were recorded for NEOSORB® Plus in a mean of 95.8% of cases compared with 75.0% of cases for NEOSORB® (*P =* 0.041) (Fig. 3).

### Secondary endpoints

The results relative to the secondary endpoints, namely specific intraoperative handling characteristics, are shown in Figs. 2 and 3. “Very good” or “excellent” scores were recorded for NEOSORB® Plus in 98.5% of cases compared with 69.7% of cases for NEOSORB®. The scores for all specific intraoperative suture handling measures (ease of passage, first-throw knot holding, knot tie-down smoothness, knot security, surgical hand memory, and degree of fraying) in the NEOSORB® Plus group were higher overall than those of the NEOSORB® group, and the difference between the groups was statistically significant. In the minimally invasive surgery group, “very good” or “excellent” scores for specific intraoperative handling characteristics were recorded for NEOSORB® Plus at a rate of 97.6% compared with 75.6% for NEOSORB® (*P =* 0.041). In the open surgery group, “very good” or “excellent” scores for all specific intraoperative suture handling measures in the NEOSORB® Plus group (mean 99.4%) were higher overall than those of the NEOSORB® group (mean 64.0%). The difference between the groups was statistically significant (*P =* 0.001) (Fig. 3).

The scores for the wound healing parameters, which were similar between the two groups, are shown in Table 4. The majority of wound erythema was mild and healed quickly. Edema occurred in 51.1% of the NEOSORB® Plus group versus 59.2% of the NEOSORB® group at POD1. However, most of the edema had disappeared at POD11. Of the patients receiving perioperative antibiotics, 17.0% were in the NEOSORB® Plus group and 18.4% were in the NEOSORB® group. On POD11, 6.4% of the NEOSORB® Plus group versus 4.1% of the NEOSORB® group had taken antibiotics for various reasons, whereas by POD30, none of the patients were taking antibiotics. The total number of cases of skin infection, seroma, and suture sinus within 30 days of after surgery was five in the NEOSORB® Plus group and two in the NEOSORB® group. The differences in these events between the two groups were not statically significant (*P* > 0.05).

**Table 4:** Wound healing scores and laboratory finding

	POD 1			POD 11		
	^a^NP	^b^N	p	NP	N	p
	(n = 47)	(n = 49)		(n = 47)	(n = 49)	
Apposition n(%)	2(4.3%)	3(6.1%)	0.52	39(83.0%)	39(79.6%)	0.35
Edema n(%)	24(51.1%)	29(59.2%)	0.43	2(4.3%)	1(2.0%)	0.49
Erythema, any n(%)	42(89.3%)	38(77.6%)	0.31	7(14.8%)	4(8.2%)	0.52
Pain, any n(%)	43(91.4%)	41(83.6%)	0.50	5(10.6%)	4(8.2%)	0.55
Antibiotics n(%)	8(17.0%)	9(18.4%)	0.59	3(6.4%)	2(4.1%)	0.52
Skin temp n(%)	24(51.1%)	21(42.9%)	0.47	2(4.3%)	1(2.0%)	0.49
Infection n(%)	0(0%)	0(0%)	–	1(2.1%)	0(0%)	0.35
Seroma n(%)	0(0%)	0(0%)	–	2(4.3%)	1(2.0%)	0.49
Suture sinus n(%)	2(4.3%)	0(0%)	0.24	2(4.3%)	1(2.0%)	0.49
Laboratory data						
WBC (10^3^/μl)	11.21 ± 0.42	10.83 ± 2.74	0.51	7.33 ± 1.92	7.31 ± 1.69	0.95
ANC(10^3^/μl)	8.40 ± 2.78	8.05 ± 2.78	0.55	4.46 ± 1.65	4.47 ± 1.49	0.97
AST(IU/l)	28.02 ± 18.57	26.72 ± 16.64	0.71	22.35 ± 10.62	20.50 ± 9.58	0.37
ALT(IU/l)	27.59 ± 27.40	25.68 ± 21.86	0.70	28.63 ± 18.90	26.60 ± 16.09	0.57
CRP(mg/dl)	3.59 ± 4.74	4.10 ± 5.76	0.64	0.65 ± 1.12	0.84 ± 1.38	0.47

*CRP* C-reactive protein, *WBC* white blood cell count, *ANC* absolute neutrophil count, *AST* aspartate aminotransferase; Alanine transaminase

^a^NP, NEOSORB® Plus; ^b^N, NEOSORB®

### Adverse events

Cumulative adverse events were reported in 8.5% of patients treated with NEOSORB® Plus and in 6.1% of patients treated with NEOSORB® (Table 5). Four patients experienced wound complications and two of these required wound revision. One patient in the NEOSORB® group was diagnosed with pre-renal azotemia. Overall, none of the adverse events was device-related, and there was no difference between the treatment groups.

**Table 5:** Cumulative adverse events

		Adverse events, n, (%)			
		Any	Serious	Requiring surgery	Device-related
Open surgery	^a^NP (*n* = 23)	2 (8.7)	0	0	0
	^b^N (*n* = 25)	3 (12.0)	2 (8.0)	1 (4.0)	0
Minimally invasive surgery	NP (*n* = 24)	2 (8.3)	1 (4.2)	1 (4.2)	0
	N (n = 24)	0	0	0	0
All patients	N (*n* = 96)	7 (7.3)	3 (3.1)	2 (2.1)	0

^a^NP, NEOSORB® Plus; ^b^N, NEOSORB®

### Certificate of non-inferiority

**Fig. 4:**
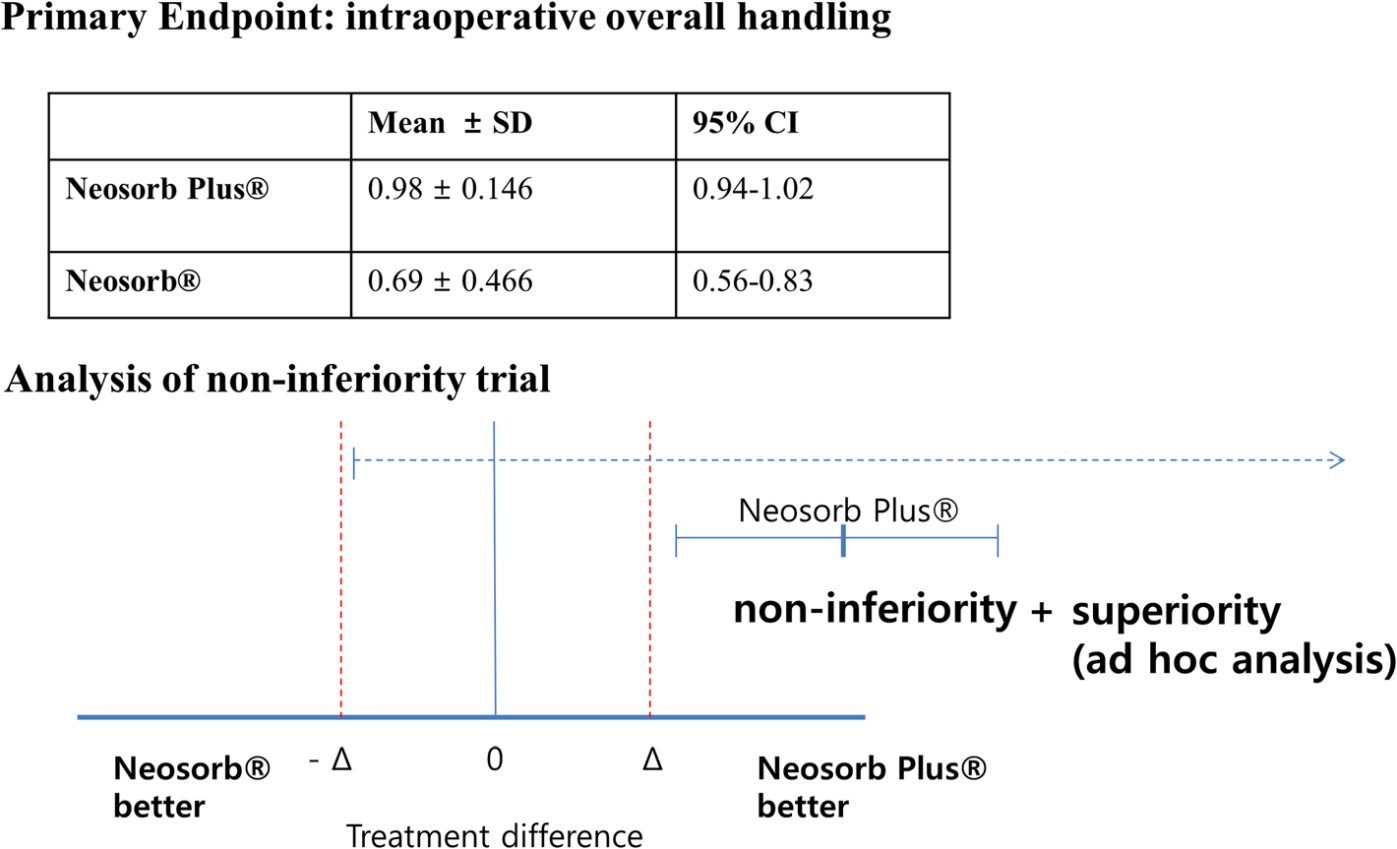
This non-inferiority threshold is the maximum allowable excess of outcome events arising from the Neosorb® Plus compared to the Neosorb®

The lower value of the two-sided 90% CI of the mean difference was within the pre-specified non-inferiority margin, which demonstrated the non-inferiority of the test compared to the control. Furthermore, the test was superior to the control, based on our ad-hoc analysis using a chi-square test (Fig. 4).

## Discussion

Recently, the increased use of minimally invasive surgery has resulted in a decrease in the incidence of SSIs. This may be attributable to the smaller incision size, earlier mobilization, reduction in postoperative pain, better preservation of immune system function, and decreased use of central venous catheters of minimally invasive procedures [16]. Nevertheless, SSI still poses a threat to patient health. SSI occurs in 2 to 3% of procedures performed in the USA and the majority (60%) of SSIs are confined to the incision site [17, 18]. In 2014, the Korean Surgical Site Infection Surveillance (KOSSIS) reported that SSI rates after colectomy and proctectomy in Korea were as high as 10.15 and 13.54%, respectively [19].

Numerous factors have been linked to elevated risk of SSI, including patient-related factors such as age, sex, lifestyle, body mass index, pre-existing infection, diabetes, comorbidities, and surgical history; and procedure-related factors such as the type of surgery, pre-surgical preparation, management of infected or colonized surgical equipment, and antimicrobial prophylaxis [20, 21]. In particular, suture knots may be a major repository of bacteria that can contaminate wounds, because they cause scarring that can promote reproduction and replication of bacteria, ultimately leading to SSI. The most common putative organisms in SSI include *Staphylococcus aureus (S. aureus)*, *Staphylococcus epidermidis (S. epidermidis)*, methicillin-resistant *S. aureus* (MRSA), and methicillin-resistant *S. epidermidis* (MRSE) [20, 22]. Therefore, inhibiting the proliferation of these organisms at the surgical site may reduce the incidence of SSI.

The suture knot is considered to be a major site of colonization of bacteria in a wound. Thus, polyglactin-910 and polydioxanone sutures coated with triclosan have been developed to confer antimicrobial activity against the most common pathogens in suture materials [2, 23]. Several clinical studies of various cohorts of surgical patients have examined whether triclosan-coated sutures effectively decrease the rate of surgical site infections [24–26]. Two recent meta-analyses demonstrated that they do in fact exert a positive effect on SSI reduction [27, 28]. However, bacterial resistance to triclosan has increased and warnings of potential pathogen selections are being emphasized [6, 7].

Segers et al. showed that CHX is effective against a broad spectrum of relevant pathogens including clinically problematic bacteria like *S. aureus* [29], and CHX has already been approved for use in a variety of medical applications such as medical device coatings, a skin antiseptic, and an oral antiseptic [30–34]. Andreas et al. evaluated novel CHX coatings for antimicrobial surgical sutures in vitro [35] and demonstrated their high antimicrobial efficacy against *S. aureus*. In particular, CHX-coated sutures with an 11 μg/Cm concentration were shown to have acceptable cytotoxicity according to ISO 10993–5 standards and simultaneously high antimicrobial protection over several days. Hence, such coated sutures can be a viable alternative to prophylactic sutures in cases of increased risk of SSI.

To our knowledge, this is the first study to evaluate the safety and efficacy of CHX-coated sutures compared with a conventional suture in humans. The incidence of adverse events between the groups was not different, and no adverse event was deemed related to the suture material in this trial. The findings demonstrated that NEOSORB® Plus was not inferior to a conventional suture in terms of overall intraoperative handling. In addition, there was no significant difference in wound healing outcomes between the two types of sutures. With regard to the wound healing parameter assessments, a high incidence of erythema and wound site pain at POD1 was seen in this study in both groups, though the erythema was generally mild and the lesions healed within one or two days. Likewise, most patients who complained of surgical wound pain showed improvement in only a few days. We consider the high incidence of surgical wound pain and erythema to be attributable to our rigorous assessment.

In the present study, NEOSORB® Plus was not inferior to NEOSORB® in overall handling compared to open surgery and laparoscopic surgery wounds. In particular, there were statistically significant differences between the NEOSORB® and the NEOSORB® Plus group in some subjects. Since NEOSORB® Plus is a newly developed product, it is likely that the needle of the instrument and the coating of the thread have been upgraded since the design of the traditional suture.

There are some limitations to the present study. First, this was a single-blinded study, where the patients did not know the group to which they were assigned. However, potential bias may have occurred because surgeons who assessed the intraoperative suture handling characteristics were not blinded. To overcome this limitation, six surgeons, with the exception of the surgeon most closely responsible for the analysis and design of the study (BST), assessed the intraoperative suture handling characteristics. Second, all of the surgical interventions in this trial were clean or clean-contaminated elective surgeries, as are the majority of urologic surgeries. In clean or clean-contaminated surgery, SSI risk is generally minimal and originates only from contaminants in the operation room environment or from the surgical team, or most commonly from skin colonists. Therefore, we concluded that NEOSORB® Plus showed no significant differences compared to traditional sutures in terms of the wound healing assessment in this study. In addition, the aim of this trial was to provide evidence for the safety and efficacy of NEOSORB® Plus, and to establish that the effectiveness of the new suture does not fall below a pre-stated non-inferiority margin. To demonstrate that NEOSORB® Plus is actually superior to the traditional suture in terms of SSI, a larger sample of participants is needed. However, since CHX did not show a negative influence on wound healing and there were no issues with the physical properties of NEOSORB® Plus in this trial, we were able to at least recognize the overall safety and feasibility of NEOSORB® Plus. In addition, further studies are expected to validate the antibacterial potential of NEOSORB® Plus in the context of other types of surgery and contaminated surgeries. Third, at the beginning of this study, a 2% dropout rate was expected, but by the end of the study a 4% dropout occurred. This may constitute an additional limitation, in that the power can be lowered. However, the sample size was sufficient to demonstrate the non-inferiority, as shown in Fig. 4, likely because the power was stronger than expected when we established our hypothesis.

As mentioned above, the original coated surgical suture, the coated PGLA910 suture with triclosan (VICRYL® Plus, Ethicon, NJ) is widely used in a variety of surgery departments. Numerous studies in vivo and in vitro have shown that triclosan-coated sutures are associated with a significantly lower risk of SSI than uncoated sutures [5]. In order to demonstrate the efficacy of NEOSORB® Plus as an alternative choice for SSI prevention, future trials with larger sample sizes are needed to accurately compare the efficacy and safety of CHX-coated sutures with that of triclosan-coated sutures.

## Conclusion

Our results indicate that the NEOSORB® Plus antibacterial suture (coated PGLA910 suture with CHX) is not inferior to the traditional suture in terms of intraoperative handling and wound healing. Further large, prospective clinical trials are warranted to validate our findings and to evaluate the potential of reduced SSI in different surgical contexts.

## Additional file

Additional file 1: Raw data of study; Raw data of Comparison Between NEOSORB Plus and NEOSORB. (XLSX 39 kb)

## Abbreviations

CHX: Chlorhexidine
POD: Postoperative dayKOSSIS: Korean Surgical Site Infection Surveillance
SSI: Surgical site infections
